# Ectopic Expression of *Aeluropus littoralis* Plasma Membrane Protein Gene *AlTMP1* Confers Abiotic Stress Tolerance in Transgenic Tobacco by Improving Water Status and Cation Homeostasis

**DOI:** 10.3390/ijms18040692

**Published:** 2017-03-24

**Authors:** Walid Ben Romdhane, Rania Ben-Saad, Donaldo Meynard, Jean-Luc Verdeil, Jalel Azaza, Nabil Zouari, Lotfi Fki, Emmanuel Guiderdoni, Abdullah Al-Doss, Afif Hassairi

**Affiliations:** 1 Plant Production Department, College of Food and Agricultural Sciences, King Saud University, P.O. Box 2460, 11451 Riyadh, Saudi Arabia; walid.brm3@gmail.com (W.B.R.); aaldoss@ksu.edu.sa (A.A.-D.); 2 Biotechnology and Plant Improvement Laboratory, Centre of Biotechnology of Sfax, University of Sfax, B.P 1177, 3018 Sfax, Tunisia; raniabensaad@gmail.com (R.B.-S.); azazajalel@yahoo.fr (J.A.); nabil.zouari@gmail.com (N.Z.); 3 CIRAD-UMR AGAP (Centre de Cooperation Internationale en Recherche Agronomique pour le Developpement), Avenue Agropolis, 34398 Montpellier CEDEX 5, France; donaldo.meynard@cirad.fr (D.M.); jean-luc.verdeil@cirad.fr (J.-L.V.); emmanuel.guiderdoni@cirad.fr (E.G.); 4 Laboratory of Plant Biotechnology Applied to Crop Improvement, Faculty of Sciences of Sfax, University of Sfax, B.P 802, 3038 Sfax, Tunisia; lotfifki@yahoo.fr

**Keywords:** drought and salt tolerance, *Aeluropus littoralis*, *AlTMP1*, plasma membrane protein, transgenic tobacco

## Abstract

We report here the isolation and functional analysis of *AlTMP1* gene encoding a member of the PMP3 protein family. In *Aeluropus littoralis*, *AlTMP1* is highly induced by abscisic acid (ABA), cold, salt, and osmotic stresses. Transgenic tobacco expressing *AlTMP1* exhibited enhanced tolerance to salt, osmotic, H_2_O_2_, heat and freezing stresses at the seedling stage. Under greenhouse conditions, the transgenic plants showed a higher level of tolerance to drought than to salinity. Noteworthy, *AlTMP1* plants yielded two- and five-fold more seeds than non-transgenic plants (NT) under salt and drought stresses, respectively. The leaves of *AlTMP1* plants accumulated lower Na^+^ but higher K^+^ and Ca^2+^ than those of NT plants. Tolerance to osmotic and salt stresses was associated with higher membrane stability, low electrolyte leakage, and improved water status. Finally, accumulation of *AlTMP1* in tobacco altered the regulation of some stress-related genes in either a positive (*NHX1*, *CAT1*, *APX1*, and *DREB1A*) or negative (*HKT1* and *KT1*) manner that could be related to the observed tolerance. These results suggest that *AlTMP1* confers stress tolerance in tobacco through maintenance of ion homeostasis, increased membrane integrity, and water status. The observed tolerance may be due to a direct or indirect effect of *AlTMP1* on the expression of stress-related genes which could stimulate an adaptive potential not present in NT plants.

## 1. Introduction

Crop yield reduction due to the direct effect of drought, salt, and cold stresses was estimated as high as 70% [1]. These stresses affect plant growth and development through osmotic stress, nutrient imbalance and the toxic effects caused by an excess of Na^+^ and Cl^−^ ions [2]. Plants can use many mechanisms to alleviate abiotic stresses such as the production of antioxidants and transport or compartmentalization of toxic Na^+^. The living cells need to maintain their intracellular ion and osmotic homeostasis, which are essential for the activities of many enzymes involved in different physiological processes. Indeed, cells must be able to preserve a high concentration of K^+^ and low concentration of Na^+^ in the cytosol [3]. In the presence of high salinity in the soil, it was shown that plants use mechanisms to maintain ion homeostasis by compartmentalizing the Na^+^ into vacuoles. In addition, plants adjust their osmotic balance by accumulating osmolytes (betaine, glycine, and proline), maintaining succulence and secreting salt [4,5]. These functions are carried out by a battery of proteins that are classified as pumps, carriers, and channels. At the cell level, plants use ion channels and ion transporters to maintain the appropriate concentration of Na^+^, K^+^, and Ca^2+^ in the cell through active and diffusion mechanisms of ion transport (reviewed in [6]). In fact, some genes such as *NHX*-type antiporters have been isolated and identified to be responsible for removing Na^+^ from the cytoplasm. This mechanism can be performed either by the exclusion of Na^+^ from plant cells (plasma membrane group: *SOS1* (*NHX7*) from *Arabidopsis*) or by the sequestration of Na^+^ in vacuoles (vacuole group: *NHX1–NHX4* from *Arabidopsis*) [7]. However, other genes have been identified which encode plasma membrane proteins performing the Na^+^ influx into the plant like *TaHKT2.1* in wheat [8] and *AtHKT1.1* in *Arabidopsis thaliana* [9]. *TaHKT2.1* was shown to co-transport K^+^ and Na^+^ in yeast and *Xenopus oocytes* [8]. During the last decade, genes responsible for ion transport were identified. These include the nonselective cation channels (NSCCs), ion transporters and membrane-potential modulators (such as a proton pump) [10,11]. The NSCCs can be divided into: cyclic-nucleotide-gated NSCCs (CNGSs), amino-acid gated NSCCs (AAG-NSCCs) and reactive-oxygen-species-activated (ROS) [10].

A small hydrophobic peptide (55 amino acids) encoded by the *PMP3* gene was suggested to be involved in preventing Na^+^ entry into the cells [12]. This plasma membrane protein modulates the membrane potential in yeast [12,13]. Due to membrane hyperpolarization, the *Δpmp3* yeast mutant is sensitive to both Na^+^ and the cationic hygromycin B [12]. PMP3 are small plasma membrane proteins which are highly conserved in bacteria, yeast, nematode and plants [14]. Many homologs to PMP3 were found in barley [15], rice [16], wheat [17], maize [3], red sage [18], *Arabidopsis* [19] and the halophyte *Aneurolepidium chinense* [20]. The transcription of all these genes was shown to be induced by low temperature, salinity, and H_2_O_2_ [15,16,19,20]. Proteins of the PMP3 family contain two highly hydrophobic domains, linked together by a short loop, and appear to be localized in the cell membrane [21]. Using the TMHMM server (TMHMM 2.0), it was shown that both the N- and C-termini of wheat WPI6 protein (PMP3 homolog) are located in the membrane whereas the internal short loop is cytoplasmic [17]. Several analyses of complementation in yeast *pmp3* mutants have shown that the expression of many plant homologs such as *OsLTi6a*, *OsLTi6b* from *Oryza sativa*, *AtRCI2a*, *AtRCI2b* from *A. thaliana*, *AcPMP3.1* from *A. chinese*, *PutPMP3.1*, *PutPMP3.2* from *Puccinellia tenuiflora*, *ZmPMP3.1* from *Zea mays*, *MsRCI2A* from *Medicago sativa*, and *MtRCI2*(A–C) from *Medicago truncatula* could functionally complement the salt sensitivity resulting from *PMP3* deletion [3,22,23]. In *Arabidopsis*, the disruption of *AtRCI2a* led to salt sensitivity due to the over-accumulation of Na^+^ in the cells [24]. Furthermore, transgenic *Arabidopsis* overexpressing *AtRCI2a* showed an enhanced salt-tolerant phenotype by decreasing Na^+^ uptake [25]. It was also shown that overexpression of *Musa paradisica* *MpRCI* in the *AtRCI2a* knockout mutant increased Na^+^ tolerance and K^+^ sensitivity under NaCl or KCl treatments, respectively. This result suggests that *MpRCI* can affect the Na^+^/K^+^ flux in the plant [26]. On the other hand, overexpression of *ZmPMP3.1* in *Arabidopsis* enhanced plant growth under salt stress by decreasing the oxidative cell damage and possibly regulating ion homeostasis [3]. More recently, it has been shown that over-expression of *MsRCI2A* in alfalfa resulted in improved salt tolerance [22]. The overexpression of *OsLTI6b* in rice enhanced its tolerance to cold stress but had no effect on salinity or drought tolerance [27]. On the other hand, overexpression of *OsRCI2–5* (sharing 60% sequence identity with *OsLti6b*) increased drought tolerance [28]. Altogether, these data suggest that PMP3 family proteins play an important role in maintaining intracellular ion homeostasis, membrane potential, and membrane organization. Despite high similarities between *PMP3* genes, different studies reported that these genes are not functionally equivalent.

Although the majority of plants cannot thrive in the presence of high concentration of salt in the soil, the extremophile halophytes can grow and reproduce in these conditions. These halophytes can be used as valuable model plants and genetic resources to decipher the molecular, biochemical and physiological mechanisms of salt tolerance in plants. This strategy will contribute to advance the improvement of salt tolerance in economically important crops. Indeed, the identification of the key genes and promoters from these extremophiles could be used to engineer plant abiotic stress tolerance either by overexpressing, silencing or editing [29]. Wild relatives of cultivated species are also a hopeful alternative source of tolerance genes: as an example, a novel stress-inducible membrane protein TdicTMPIT1 predicted to be localized in the chloroplast was recently isolated from drought tolerant wild emmer wheat and not present in modern wheat cultivars, Kiziltan, has been shown to be an interesting candidate with a putative role in stress signaling [30]. To eventually improve the tolerance to abiotic stresses in cereals, we are using the halophyte grass *A. littoralis* as a source of candidate genes and their promoters. This extremophile previously described by [31], is a perennial C4 grass, can secrete NaCl by salt glands and has (2*n* = 2*X* = 10) chromosomes with an estimated genome size of 349 Mega bp. To decipher the mechanisms behind salt tolerance in this halophyte plant at the genomic level, we have isolated 492 ESTs (expressed sequence tag) from a differentially expressed cDNA library prepared by suppression subtractive hybridization (SSH) using salt-stressed roots [31]. These ESTs shared significant similarities with sequences from rice, maize and sorghum [31] and the functional analysis of their full-length genes in tobacco can allow the identification of the key candidate genes to be used for crop stress tolerance improvement. As a demonstration of this strategy, we previously reported the isolation of the *AlSAP* gene and its promoter [32,33]. The constitutive expression of *AlSAP* in tobacco, wheat, and rice has resulted in enhanced tolerance to drought, salinity, cold, heat, and oxidative stresses [32,34,35].

To decipher the efficient regulation systems for ion homeostasis and osmoregulation under salt stress developed by the halophyte grass *A. littoralis*, we isolated and characterized a gene (*AlTMP1*, KY321744) encoding a PMP3 protein. In *A. littoralis*, *AlTMP1* was shown to be induced by abscisic acid (ABA), cold, salt and osmotic stresses. We have found that the expression of the *AlTPM1* gene in tobacco improved tolerance more efficiently to continuous drought than to salinity under greenhouse conditions. In addition, these transgenic tobacco plants showed significantly improved tolerance to heat and cold stresses. Interestingly, *AlTMP1* plays a role in maintaining high membrane stability. Finally, *AlTMP1* accumulation was shown to alter the expression some stress related genes under both control and stress conditions and likely primes the plants to tolerate stresses.

## 2. Results

### 2.1. Isolation and Sequence Analysis of the AlTMP1 Gene

**Figure 1:**
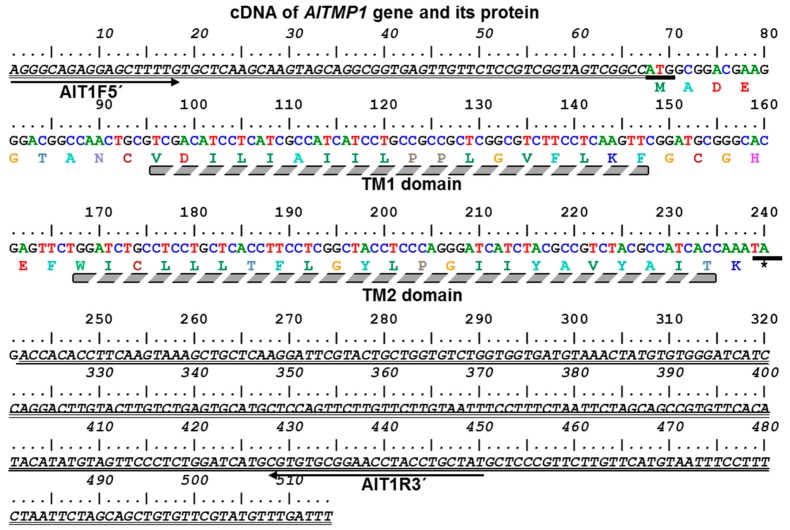
Full-length cDNA sequence and predicted protein sequence of *AlTMP1*. The amino acid sequence is shown in single code letters beneath the cDNA sequence. Translational start codon (ATG) and stop codon (TAG) are underlined. The 5′ and 3′ non-coding sequences are double underlined. The conserved transmembrane domains (TM1 and TM2) are highlighted in gray. The sequence was analyzed with BioEdit 7.2.5 programs and the TMHMM 2.0 server. The * design the stop codon terminating AlTMP1 protein synthesis.

The differentially expressed *AlTMP1* full-length cDNA clone (accession number in GenBank: KY321744) was isolated following the screening of an SSH cDNA library previously prepared [31] from roots of *A. littoralis* plants stressed with 300 mM NaCl during 15 days. The sequence analysis revealed that *AlTMP1* cDNA is 514 bp long, including a complete open reading frame (ORF) of 174 bp with 5′-UTR and 3′-UTR regions of 67 and 273 bp, respectively (Figure 1). The comparison between *AlTMP1* cDNA and its amplified genomic sequence revealed that the gene contains two exons separated by one intron of 109 bp (Figure S1). *AlTMP1* encodes a 57-amino-acid protein with a predicted molecular weight of 6.23 kDa, a pI of 4.75 (http://web.expasy.org/compute_pi/) and contains a conserved domain with high homology (*E*-value: 9.00 *e*^−14^) to the pfam01679 (https://www.ncbi.nlm.nih.gov/Structure/cdd) (Figure S2A). The search for the presence of predicted transmembrane helices in AlTMP1 protein using the TMHMM 2.0 server [36] revealed the presence of two α-helices transmembrane domains TM1 (from aa10 to aa27) and TM2 (from aa34 to aa56) which are separated by one short loop of six amino acids (Figure S2B). In addition, the TMHMM 2.0 analysis predicted that the N-terminal of 9aa length is outside the membrane (Figure S2B). Moreover, by analyzing the hydropathy using the software “protparam” (http://web.expasy.org/protparam/), the AlTMP1 polypeptide seems to be highly hydrophobic as its predicted grand average of hydropathicity (GRAVY) value is 1.225 (Figure S2C).

**Figure 2:**
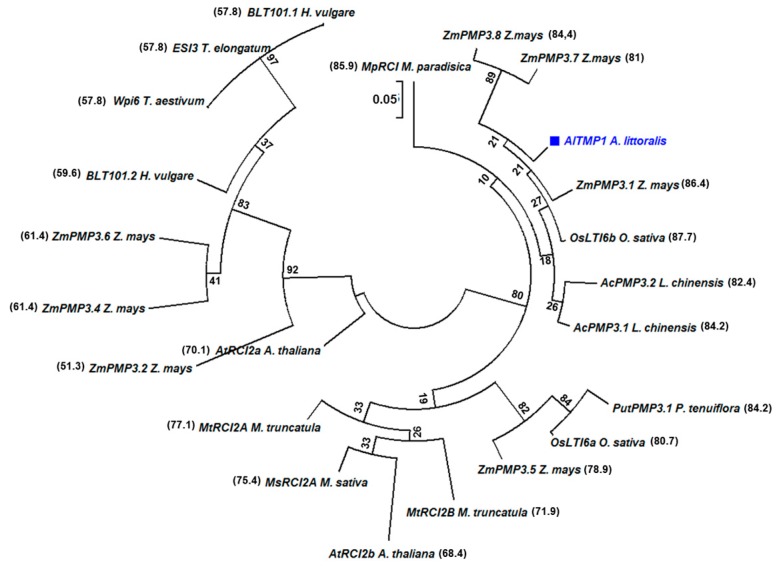
Phylogenetic analysis of AlTMP1 and its homologs from different plants. The phylogenetic tree was constructed with the full-length amino acid sequences of PMP3 family proteins in *Z. mays*, *O. sativa*, *P. tenuiflora*, *H. vulgare*, *T. aestivum*, *Thinopyrum elongatum*, *L. chinensis*, *Musa*, *A. thaliana*, *M. truncatula* and *M. sativa*. The MEGA 6 program and the Neighbor-joining method with 1000 replicates were used to generate the phylogenetic tree. Numbers are bootstrap values. The percentages of identity between *AlTMP1* gene and different PMP3 proteins are indicated between brackets (%). The *AlTMP1* gene isolated in this study is in blue font.

The search of NCBI database using blastp revealed a significant high identity between *AlTMP1* and other genes encoding PMP3 proteins isolated from monocot and dicot plants (*ZmPMP3.1* to *ZmPMP3.8* from *Z. mays* [3], *OsLti6a* and *OsLti6b* from *O. sativa* [16], *AcPMP3.1* and *AcPMP3.2* from *Leymus chinense* [20], *PutPMP3.1* and *PutPMP3.2* from *P. tenuiflora* [23], *BLT101.1* and *BLT101.2* from *Hordeum vulgare* [15], *WPI6* from *Triticum aestivum* [17], *ESI3* from *Lophopyrum elongatum* [37], *MpRCI* from *Musa paradisiaca* [26,38], At*RCI2a* and At*RCI2b* from *A. thaliana* [39], *MsRCI2A* from *M. sativa* [22], *MtRCI2A* and *MtRCI2B* from *M. truncatula* [22]) as well as to the *PMP3* from *Saccharomyces cerevisiae* [12] (Figure S3). Comparison of the amino acid sequences of 25 *PMP3* genes isolated from a monocot and dicot plants revealed that the transmembrane domains were highly conserved, in particular, the pfam01679 domain. However, the size of these genes was different due to the variable lengths of the C- and N-terminal regions (Figure S3). To study the evolutionary relationships between *AlTMP1* and its homologs, a phylogenetic tree was constructed by using the MEGA 6 program and the Neighbor-joining method with 1000 replicates. Six highly homologous genes to the *AlTMP1* were predicted (Figure 2). The two nearest homologs were found in *O. sativa*, *OsLTI6b* (AAT37942.1) and *Z. mays*, *ZmPmP3.1* (NP001107634), with a high identity percentage of 87.7% and 86.4%, respectively (Figure 2). Four others homologs were found in *Z. mays* (*ZmPmP3.7* and *ZmPmP3.8*) and *Leymus chinensis* (*AcPmP3.1* and *AcPmP3.2*) with a lower identity ranging between 81% and 84%, whereas, for other *PMP3* genes from the remaining dicots and monocots plants, the percentage identity was much lower, ranging from 60% to 80%. Apart from the plant homologs, the *AlTMP1* showed 31% identity to the yeast *Pmp3p* gene.

### 2.2. AlTMP1 is a Plasma Membrane Protein

**Figure 3:**
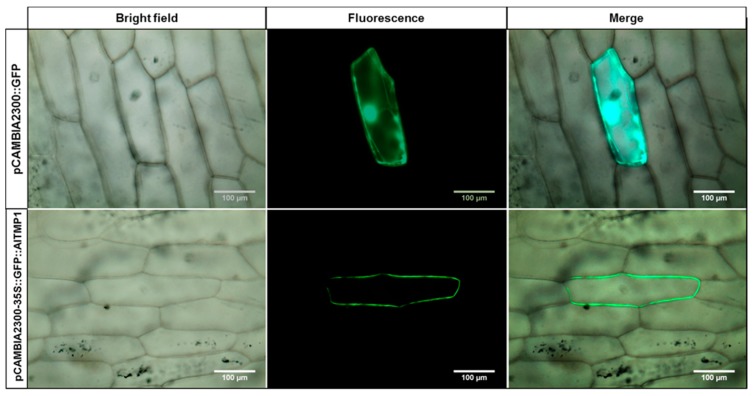
Subcellular localization of AlTMP1::GFP in onion epidermal cells. 35S::GFP and 35S::GFP::AlTMP1 fusion constructs were transiently expressed in onion epidermal cells using particle bombardment. The onion epidermal cells expressing GFP were set as control. The fluorescent signals were examined by confocal microscopy 36 h after bombardment. The images of onion epidermal cells expressing 35S::GFP::AlTMP1 fusion were taken in a superposition of bright and fluorescence vision (**right**), fluorescence field vision (**center**), and bright light vision (**left**). Bar = 100 µm.

By using the hydrophobicity plotting or the algorithms TMHMM, it was predicted the presence of two conserved putative transmembrane domains in AlTMP1 protein. The green fluorescent protein (GFP) (pCAMBIA2300::GFP) and the GFP::AlTMP1 fusion proteins (pCAMBIA2300::GFP::AlTMP1) were transiently expressed in onion epidermal cells, and their subcellular localizations were visualized by confocal microscopy. In control cells transformed with the GFP construct, the fluorescence was observed in the cytoplasm, nucleus, and cell membrane, while, in cells expressing the GFP::AlTMP1 fusion gene, the GFP signal was observed only in the cell membrane, suggesting that AlTMP1 was localized to the plasma membrane (Figure 3).

### 2.3. AlTMP1 Transcription Responds to Abiotic Stresses

**Figure 4:**
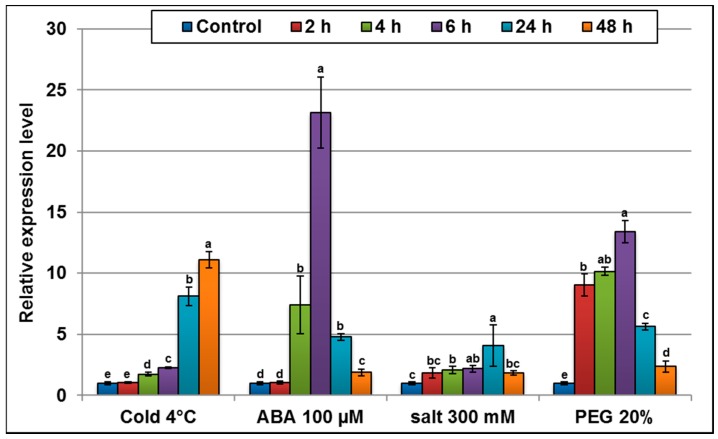
qRT-PCR analysis of *AlTMP1* transcripts in *A. littoralis* plants challenged with various stresses. Two-month-old *A. littoralis* plants were treated with 300 mM NaCl, 4 °C, 20% PEG 8000 and 100 µM abscisic acid (ABA) for 2, 4, 6, 24 and 48 h. The rRNA18s gene was used as an internal control. Data from qRT-PCR experiments were analyzed according to the 2^−∆∆*C*t^ method. Vertical bars indicate standard deviation calculated from three replicates. Values are mean ± s.e. (*n* = 3). Means denoted by the same letter did not differ significantly at *p* < 0.05.

The *AlTMP1* transcript abundance was determined by performing qRT-PCR on total RNA isolated from *A. littoralis* plants treated with different abiotic stresses (salt, osmotic and cold) and to hormonal stress (ABA). PEG 8000 20% treatment stimulates an early accumulation of *AlTMP1* transcripts up to 10-fold higher than that in control plants, following two hours of stress application. As to the NaCl, ABA, and cold treatments, significant increases of AlTMP1 transcripts are observed only after 4 h from the beginning of stress application (Figure 4). The maximum relative expression levels of AlTMP1 transcripts were observed following ABA stress (23-fold higher than the control treatment) and PEG 20% treatment (13-fold higher than the control) after 6 h (Figure 4). These results indicate that the *AlTMP1* gene is clearly regulated by abiotic and hormonal stresses in *A. littoralis*. They also suggest that the *AlTMP1* is more sensitive to treatments imposing osmotic stress than those triggering ionic stresses and that its regulation is ABA-dependent.

### 2.4. AlTMP1 Enhances Abiotic Stress Tolerance in Transgenic Tobacco

**Figure 5:**
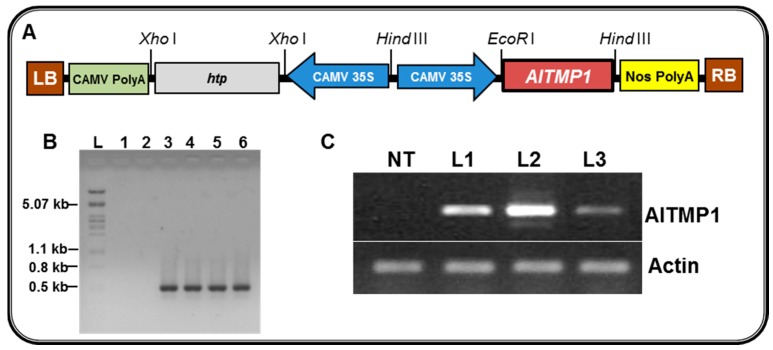
Molecular analysis of T2 transgenic tobacco lines (L1, L2, and L3) expressing *AlTMP1*. (**A**) Schematic map of the T-DNA inserted into the binary vector pCAMBIA1390::*AlTMP1*, used for tobacco transformation; LB: left border T-DNA repeat; RB: right border T-DNA repeat; *hpt*: gene codes for hygromycin phosphotransferase which detoxifies the antibiotic hygromycin B; (**B**) PCR amplification products from T2 transgenic and non-transgenic (NT) tobacco plants. Genomic DNA of NT and transgenic tobacco lines was used to amplify *AlTMP1* gene with specific primers; lane 1: ddH_2_O; lane 2: NT tobacco DNA; lanes 3–5: transgenic clones L1, L2, and L3 DNA; lane 6: pCAMBIA1390::*AlTMP1* binary vector DNA used as positive control; lane L: Lambda DNA *Pst*I Marker; (**C**) Expression pattern of the *AlTMP1* gene in transgenic lines (L1, L2, and L3). The RT-PCR analysis was performed with *AlTMP1* specific primers using the RNA isolated from the transgenic lines and NT plants. Actin amplification was used as an internal control.

To further investigate the role of *AlTMP1* in conferring tolerance to salinity, drought, and cold/heat stresses, we generated transgenic tobacco plants expressing *AlTMP1* under the control of the CaMV-35S promoter (Figure 5A). Three independent transgenic tobacco lines, accumulating *AlTMP1* transcripts at different levels were selected (L1, L2, and L3). The presence of *AlTMP1* gene in tobacco transformants was confirmed by PCR and its integration into the genome was demonstrated by sexual transmission to T1 and T2 progenies (Figure 5B). The constitutive expression of *AlTMP1* in the selected transgenic lines was evaluated by semi-quantitative RT-PCR analyses (Figure 5C). Two transgenic lines, L2 and L3, respectively, the highest and lowest *AlTMP1* expressers, were selected for stress assays under in vitro and greenhouse conditions (Figure 5C).

#### 2.4.1. Evaluation of Stress Tolerance under In Vitro Conditions

**Figure 6:**
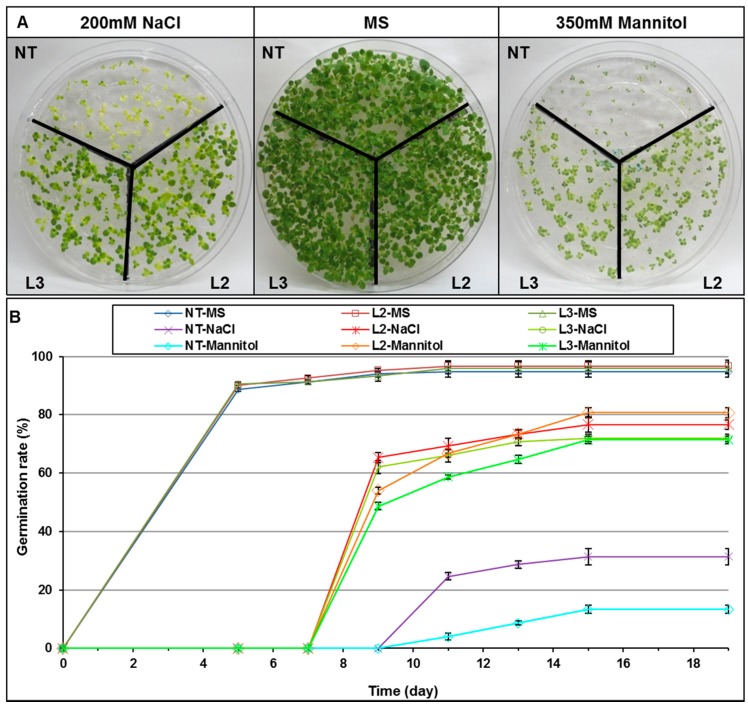
Effect of salt (NaCl 200 mM) and osmotic (mannitol 350 mM) stresses on seed germination and plant phenotype in transgenic tobacco plants expressing *AlTMP1*. (**A**) Photographs were taken two months after seed germination; (**B**) Seed germination rates of NT and transgenic plants that are grown on MS medium (control), MS supplemented with 200 mM NaCl or MS supplemented with 350 mM mannitol. Vertical bars indicate standard deviation calculated from three replicates of 100 seeds each.

The tolerance to salt (NaCl) and osmotic (mannitol) stresses of homozygous T2 seedlings of two transgenic lines, L2 and L3 were first evaluated under in vitro conditions. Seeds of non-transgenic tobacco (NT) and the two homozygous transgenic lines L2 and L3 were germinated on different MS media MS0 (control), MS0s (200 mM NaCl) and MS0m (350 mM mannitol). When using MS0, no significant difference was observed in the rate and the percentage of germination between NT and transgenic seeds, indicating that expression of *AlTMP1* gene had no effect on seed germination (Figure 6A,B). In the presence of salt (MS0s: 200 mM NaCl) or osmoticum (MS0m: 350 mM mannitol), the germination of NT seeds was delayed by six days compared to that observed in the control medium. Moreover, only 31% and 13% of NT seeds were able to germinate following 19 days of salt and osmotic stresses, respectively (Figure 6B). However, delay in seed germination of transgenic lines L2 and L3 was reduced to only four days in both MS0s or MS0m treatments (Figure 6B), with a high percentage of germination ranging from 70% to 80%. Following germination, the growth of NT seedlings stopped, and leaves appeared small (osmotic stress) or yellow (salt stress). Conversely, shoots and roots of all the transgenic seedlings continued to grow normally (Figure 6A). For the higher *AlTMP1* expressor line L2, the germination was 77% and 81% for salt and osmotic stresses, respectively (Figure 6B). This represents 2.5- and 6-fold enhancements under salt and osmotic stresses, respectively, when compared to NT (Figure 6B). All these results suggest that expression of *AlTMP1* can enhance seed germination under salt or osmotic stresses. Interestingly, the improved tolerance conferred by *AlTMP1* at the seed germination stage was higher in the case of osmotic stress than for salt stress.

**Figure 7:**
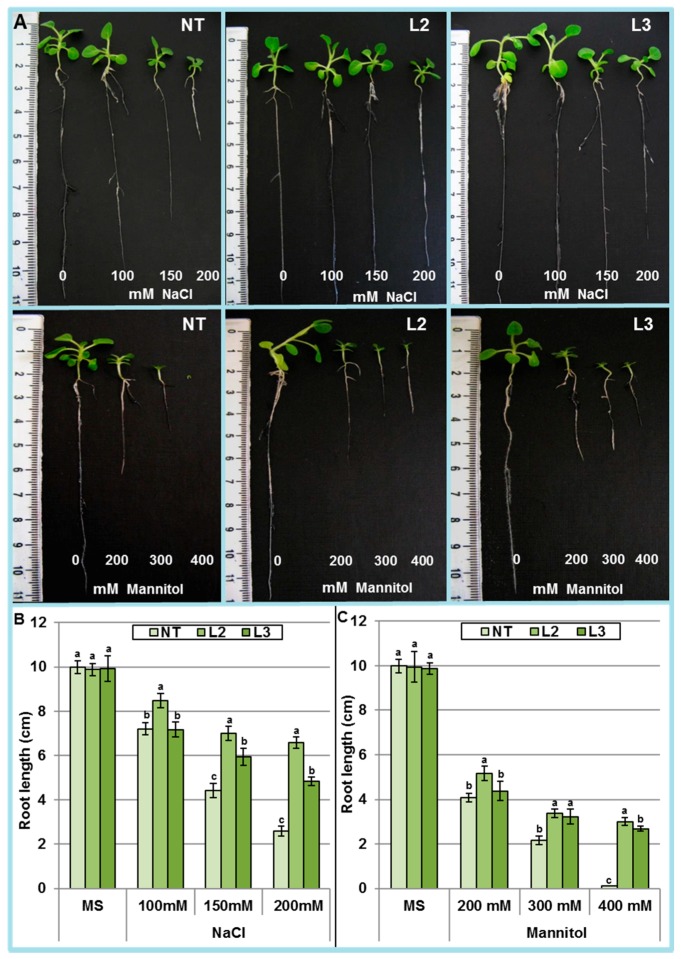
Effect of various concentrations of salt and mannitol on root elongation of non-transgenic (NT) and two *AlTMP1* transgenic tobacco seedlings. (**A**) Photographs were taken four weeks after incubation in vitro; (**B**) Root length of NT and transgenic seedlings (L2 and L3) grown on MS medium (control) or various concentrations of NaCl; (**C**) Root length of NT and transgenic seedlings (L2 and L3) grown on MS0 (control) or various concentrations of mannitol. Values are mean ± s.e. (*n* = 3). Means denoted by the same letter did not differ significantly at *p* < 0.05.

To further investigate the effect of salt and mannitol on the root growth, ten-day-old NT and transgenic seedlings germinated on MS0 were transferred to MS0, MS0s (NaCl 100, 150 and 200 mM), and MS0m (mannitol 200, 300 and 400 mM). After one month, root and shoot length of NT seedlings were strongly affected by the salt and osmotic stresses, whereas, growth inhibition was less pronounced in transgenic lines (Figure 7A). The roots of plants of the L2 line were 2.5 and 30 fold longer than those of NT under 200 mM NaCl and 400 mM mannitol, respectively (Figure 7B,C). In addition, the length of L2 roots was significantly greater than those of L3 line under these stresses. This better performance of L2 could be ascribed to the higher expression level of *AlTMP1* found in this line.

**Figure 8:**
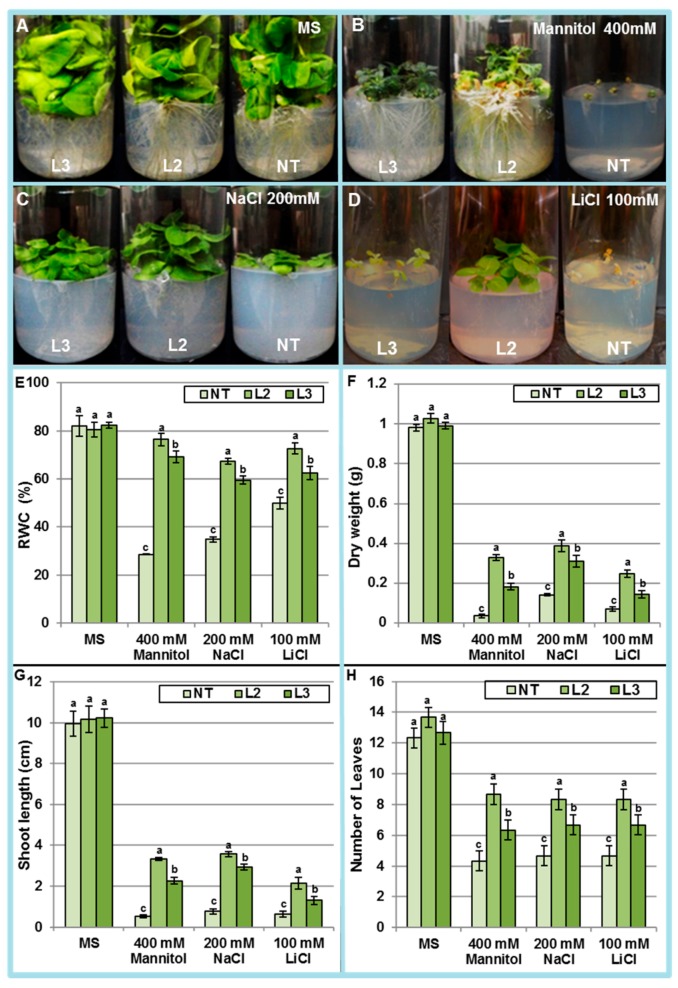
Evaluation of NT and (L2, L3) transgenic lines expressing *AlTMP1* grown on MS, mannitol, LiCl, and salt-supplemented media. (**A**–**D**) Growth comparison of NT and (L2 and L3) transgenic lines after 60 days in (**A**) MS; (**B**) 400 mM Mannitol; (**C**) 200 mM NaCl and (**D**) 100 mM LiCl; (**E**) Relative water content (RWC) of NT and (L2 and L3) transgenic tobacco lines; (**F**) Plant dry weight in NT and (L2 and L3) transgenic lines grown on MS, mannitol, LiCl, and salt-containing media; (**G**) Shoot length of NT and (L2 and L3) transgenic tobacco lines; (**H**) Number of leaves in NT and (L2 and L3) transgenic tobacco lines. Vertical bars indicate standard deviation calculated from three replications. Values are mean ± s.e. (*n* = 3). Means denoted by the same letter did not differ significantly at *p* < 0.05.

Seedlings of NT and transgenic lines after ten days of germination on MS0 were transferred to culture media imposing in vitro osmotic (mannitol 400 mM), salt (NaCl 200 mM) or ionic (LiCl 100 mM) stresses. Following two months of growth, the whole plant dry weight, the relative water content (RWC), the number of leaves, and the shoot length were evaluated (Figure 8A–H). Whereas the leaves of the NT plants had turned yellowish and wilted, leaves of the transgenic plants were still green leaves, and the shoots were relatively able to elongate (Figure 8A–D). Relative water content (RWC) is probably the most appropriate measure of plant water status. Under osmotic, salt and ionic stresses, transgenic plants retained more water in their leaves than the NT plants (Figure 8E). The RWC for NT plant was only 3%, 35% and 49% under osmotic, NaCl and LiCl stresses, respectively (Figure 8E). On the other hand, plants of the highest *AlTMP1* expressing line (L2) maintained their RWC up to 75%, 72% and 67% under osmotic, LiCl and NaCl stresses, respectively (Figure 8E). These results demonstrate that expressing of *AlTMP1* in tobacco can enhance the ability of transgenic plants to retain water up to 25 fold more than NT under osmotic stress. Along the same line, the dry weight accumulated by transgenic plants was ten- and three-fold more than that of NT under osmotic and NaCl stresses, respectively (Figure 8F). In addition, transgenic plants exhibited significantly longer shoot and a higher number of leaves than NT plants under different stresses (Figure 8G,H). These results demonstrated that *AlTMP1* gene could enhance the tolerance to NaCl, osmotic and ionic stresses. Again, the level of tolerance attained by the transgenic line seemed correlated with the level of *AlTMP1* transcript accumulation. Finally, confirming observations at germination stage, the protection conferred by *AlTMP1* appears higher in the case of osmotic stress than for NaCl and ionic stresses.

**Figure 9:**
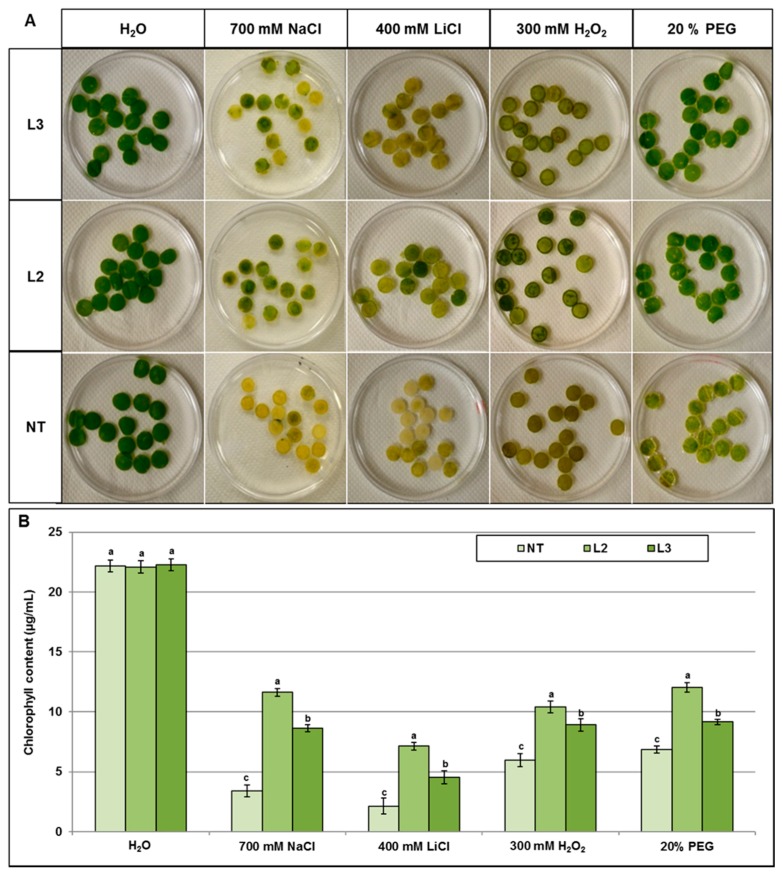
Leaf discs floating assays as an evaluation of ionic and osmotic stress tolerance in transgenic *AlTMP1* tobacco lines. (**A**) The representative picture is showing the phenotypic differences in leaf discs bleaching; (**B**) Chlorophyll content in the leaf discs of NT and two transgenic lines plants (L2 and L3) after incubation in 700 mM NaCl, 400 mM LiCl, 300 mM H_2_O_2_ and 20% PEG. Leaf discs floating in water served as experimental control. After 72 h of incubation, the chlorophyll content in the leaf discs was measured. Values are mean ± s.e. (*n* = 3). Means denoted by the same letter did not differ significantly at *p* < 0.05.

A detached leaf disc assay was performed to evaluate the chlorophyll content under stresses. This assay can be considered as a reliable index reflecting the damage of the photosynthesis process under stress. When leaf discs were floated on a NaCl 700 mM, LiCl 400 mM PEG 20% and H_2_O_2_ 300 mM solution for 72 h, discs of NT plants bleached more dramatically than those of transgenic lines (Figure 9A). These observations were verified by measuring chlorophyll content in leaf discs. When compared to control condition the NT discs retained only 15%, 20%, 27% and 31% of their chlorophyll under NaCl, LiCl, H_2_O_2_, and PEG, respectively (Figure 9B). On the other hand, the leaf discs of L2 line retained 54%, 52%, 45%, and 32% of their chlorophyll under PEG, NaCl, H_2_O_2_, and LiCl, respectively (Figure 9B). Consistent with previous observations, leaf discs of the L2 line displayed higher tolerance to chlorophyll bleaching than those of the L3 line under the different stresses. Altogether, these results support the suggestion that *AlTMP1* improves more efficiently tolerance to osmotic stress (the highest chlorophyll retention 54%) than to other stresses.

#### 2.4.2. Evaluation of Stress Tolerance under Greenhouse Conditions

##### *AlTMP1* Confers Tolerance to Cold and Heat Stresses

**Figure 10:**
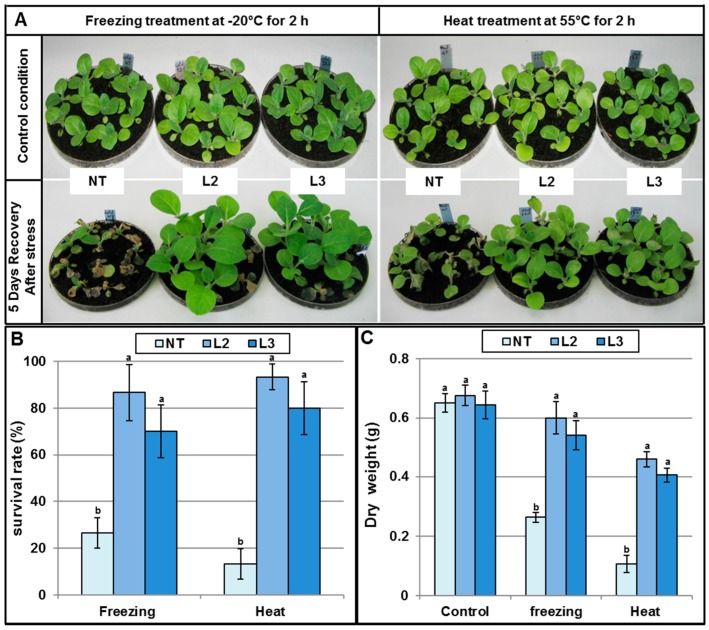
Effect of freezing and heat stresses on non-transgenic and transgenic AlTMP1 plants. (**A**) Plant phenotype of tobacco non-transgenic (NT) and transgenic plants (L2 and L3) after freezing and heat treatments followed by five-day recovery period at ambient environment; (**B**) The survival rate five days after the freezing and heat treatment as shown in (**A**); (**C**) The dry weight of NT and transgenic plants five days after the freezing and heat treatments. The experiments were repeated three times with similar results. Values are mean ± s.e. (*n* = 3). Means denoted by the same letter did not differ significantly at *p* < 0.05.

As the transcription of the *AlTMP1* gene is induced by low-temperature (4 °C), L2 and L3 transgenic lines plants along with NT were confronted to freezing (−20 °C for two h) and heat (55 °C for two h) stresses. After five days only 13% and 26% of NT seedlings were able to recover from freezing and heat stresses respectively (Figure 10A,B). On the other hand, nearly 90% of transgenic seedlings recovered following the two types of stresses (Figure 10A,B). Consequently, the dry weight of recovered transgenic plants was significantly higher than those of NT plants (Figure 10C).

##### *AlTMP1* Confers Tolerance to Continuous Salt and Drought Stresses

**Figure 11:**
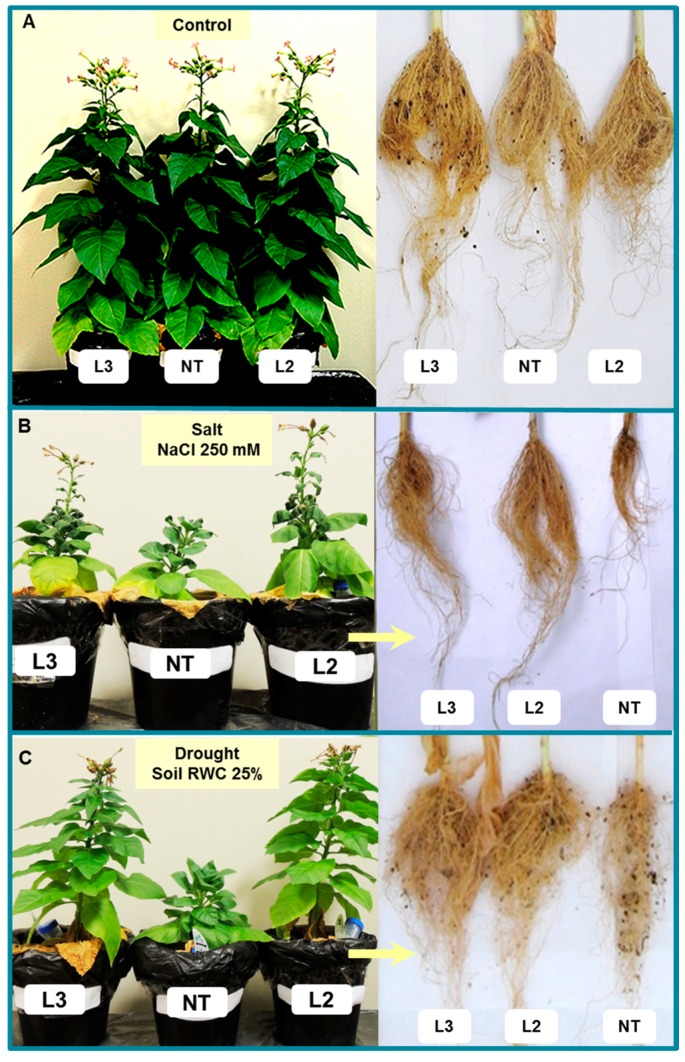
Evaluation of salt and drought tolerance of non-transgenic and *AlTMP1* transgenic tobacco plants at adult stage under greenhouse conditions. Phenotypes of NT and transgenic plants which were grown under: normal condition (**A**); continuous irrigation with a salty solution (250 mM NaCl) (**B**); and continuous drought stress (soil RWC of 25%) (**C**).

**Figure 12:**
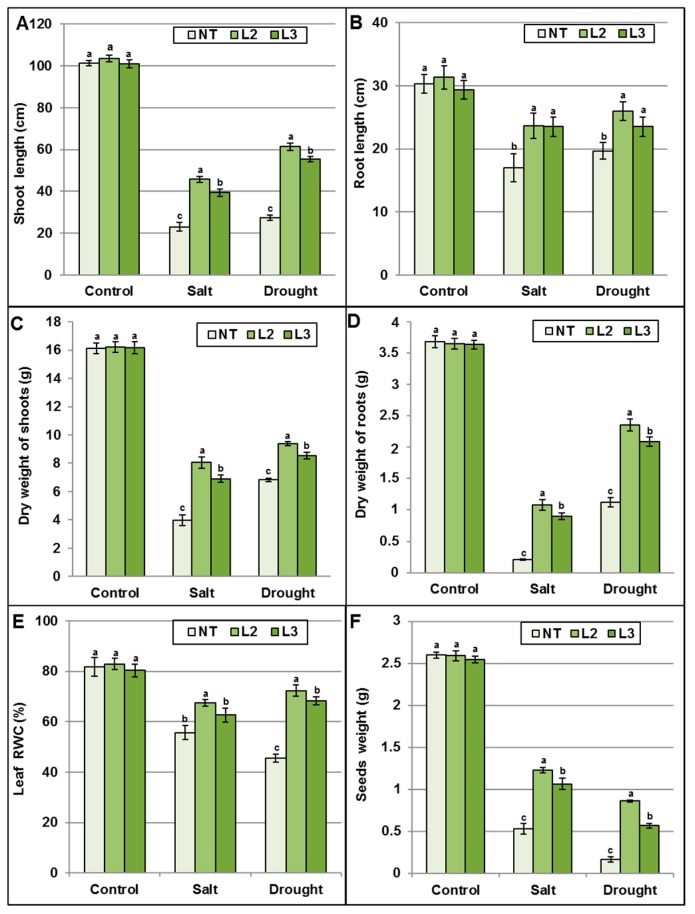
Evaluation of tolerance of non-transgenic and transgenic *AlTMP1* (L2 and L3 lines) plants subjected to continuous salt and drought stresses under greenhouse growth conditions: (**A**) Shoot length; (**B**) Root length; (**C**) Shoot Dry weight; (**D**) Root dry weight; (**E**) Leaf relative water content (RWC); and (**F**) Weight of produced seeds (g per plant). Values are mean ± s.e. (*n* = 3). Means denoted by the same letter did not differ significantly at *p* < 0.05.

**Figure 13:**
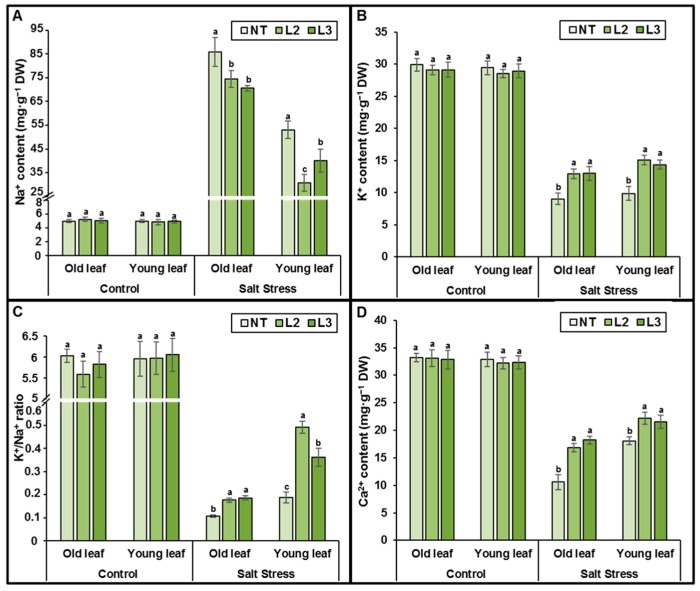
Na^+^, K^+^ and Ca^2+^ contents in young and old leaves of non-transgenic and transgenic *AlTMP1* (L2 and L3 lines) plants grown in a greenhouse under control and salt (250 mM NaCl) stress conditions. Values are mean ± s.e. (*n* = 3). Means denoted by the same letter did not differ significantly at *p* < 0.05.

**Figure 14:**
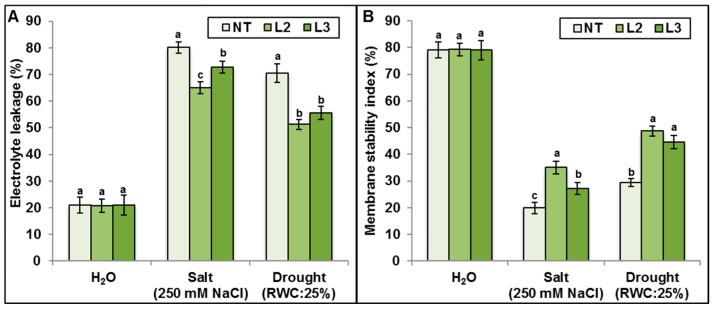
Electrolyte leakage (**A**); and membrane stability index (**B**) of non-transgenic and transgenic *AlTMP1* (L2 and L3 lines) plants grown in greenhouse conditions under salt (250 mM NaCl) stress conditions. Values are mean ± s.e. (*n* = 3). Means denoted by the same letter did not differ significantly at *p* < 0.05.

Previous in vitro assessment of *AlTMP1* transgenic tobacco lines provided a first evidence that they exhibit an enhanced tolerance to salt and osmotic stresses. We further investigated the effect of this gene on growth and seed production under control conditions, drought stress (the RWC of the soil was maintained at 25%) or salt stress (irrigation with NaCl 250 mM) until the end of the plant cycle in the greenhouse. Several critical parameters related to plant growth (plant dry weight, root and shoot length, leaf RWC, seed weight, K^+^ and Na^+^ content, membrane stability index and electrolyte leakage) were scored. These parameters were used to evaluate the salt and drought stress tolerance in T2 homozygous transgenic lines (Figure 11, Figure 12, Figure 13 and Figure 14). As shown in Figure 11A, the ectopic expression of *AlTMP1* does not affect overall plant morphology since no obvious growth or yield penalty was observed between NT and *AlTMP1* transgenic plants grown under control growth conditions. However, the performance of *AlTMP1* transgenic plants was clearly improved under salt and drought stresses. Indeed they were able to continue their growth, reach the flowering stage and set seeds while the NT plants showed a stunted phenotype (Figure 11B,C). The transgenic plants flowered at the same time either under drought stress or control conditions, while under salt stress flowers appeared with a delay of nearly 12 days. On the other hand, compared to control conditions NT flowered with a delay of 18 days under both stresses. Lengths and dry weight of roots and shoots were significantly higher in transgenic plants than in NT under both drought and salt stresses (Figure 12A–D). In addition, as shown in Figure 12E, *AlTMP1* enhances the RWC in leaves under drought and salt stresses. Indeed, the RWC was about 67%–70% in transgenic plants compared to 45%–55% in NT plants either under salt or drought conditions. Interestingly, the most striking difference between transgenic and NT plant was observed in seed production. The L2 *AlTMP1* transgenic line produced two- and five-fold more seeds than NT under salt and drought stresses, respectively (Figure 12F). The L2 line which accumulates the highest level of *AlTMP1* transcripts exhibited a significantly higher tolerance to salt and drought than the lower expressor L3 line.

The concentration of Na^+^, K^+^, and Ca^2+^ were measured in young and old leaves of the transgenic lines and NT plants following 60 days of salt treatment. No significant difference is observed in Na^+^, K^+^ or Ca^2+^ accumulation and partitioning in leaves of NT and transgenic plants under control conditions. However, a significant change is seen in the partitioning of Na^+^ and K^+^ in plants grown under 250 mM NaCl. Thus, in transgenic plants, young and old leaves exhibited significant lowest Na^+^ ion content compared to NT plants (Figure 13A). Na^+^ accumulation in young NT leaves was two-fold higher than in those of *AlTMP1* transgenic lines. In general, both transgenic lines exhibited two-fold higher concentrations of Na^+^ in old leaves than in young leaves under stress conditions. Furthermore, under control condition, all leaves of transgenic lines and NT exhibited K^+^ contents that were nearly equal. However, under salt stress condition, young leaves of transgenic lines accumulated more K^+^ and Ca^2+^ than those of NT plants (Figure 13B,D). Consequently, the transgenic plants were able to maintain a relatively high K^+^/Na^+^ ratio compared to NT plants (Figure 13C). 

No significant difference in electrolyte leakage (EL) (Figure 14A) and membrane stability index (MSI) (Figure 14B) was noticed between transgenic and NT plants grown under control conditions. However, under stress condition, transgenic lines showed significantly higher MSI and lower EL compared to control plants (Figure 14A,B). These results could explain the better capacity of transgenic lines to maintain higher osmotic adjustment and K^+^/Na^+^ ratio.

### 2.5. AlTMP1 Regulates the Expression of Some Stress-Related Genes in Transgenic Tobacco

**Figure 15:**
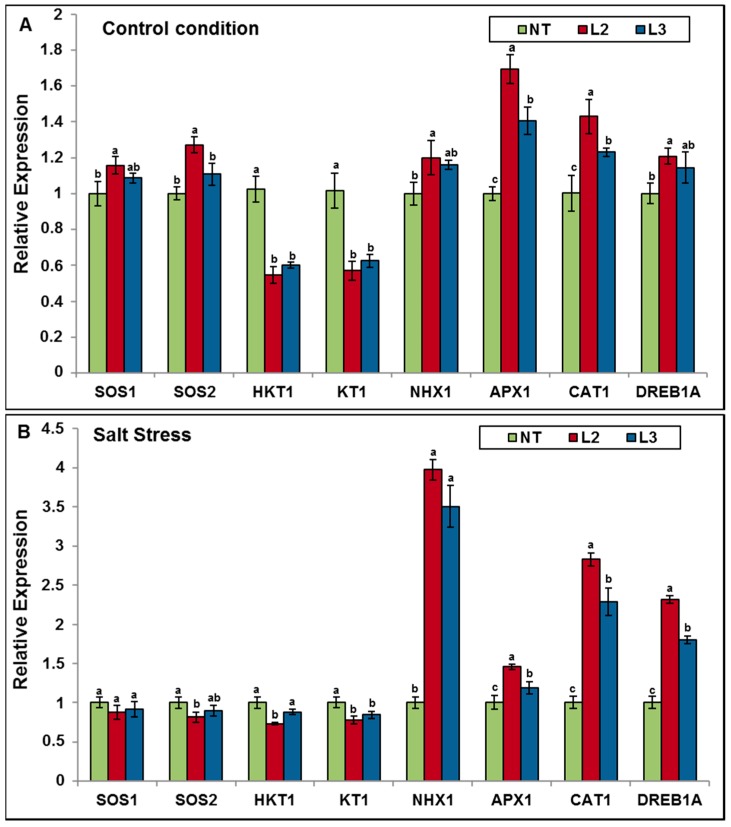
Relative expression of stress-related genes in *AlTMP1* transgenic lines (L2, and L3) and NT tobacco plants under control and salt stress conditions. Values are mean ± s.e. (*n* = 3). Means denoted by the same letter did not differ significantly at *p* < 0.05.

To further investigate the molecular mechanisms of enhanced abiotic stress tolerance afforded by the *AlTMP1* expression, real-time qRT-PCR analysis was performed on eight stress-related genes in leaves of NT and transgenic tobacco plants grown under normal and stress conditions. As shown in Figure 15A, four (*SOS2*, *DREB1A*, *APX1* and *CAT1*) and two (*HKT1* and *KT1*) out of these eight genes were significantly (*p* < 0.05) up- and down-regulated, respectively, in transgenic *AlTMP1* tobacco grown under control conditions. Under salt stress conditions, *NHX1*, *DREB1A*, *APX1* and *CAT 1* were significantly up-regulated while *KT1* and *HKT1* were down-regulated (Figure 15B). These results suggest that *AlTMP1* possibly acts as a positive or negative regulator of genes involved in ions homeostasis (*NHX1*, *SOS2*, *HKT1*, *KT1*) and antioxidant defense system (*CAT1*, *APX1*) that contributes to the enhanced salt tolerance observed in transgenic *AlTMP1* lines. Along the same line, accumulation of *AlTMP1* resulted in the up-regulation of a key transcription factor, DREB1A responsible for the induction of multiple stress tolerance genes involved in drought, high salt, and cold responses.

## 3. Discussion

Plants are known to maintain their ion homeostasis in response to salt stress by increasing the concentration of K^+^ in the cytosol with a concomitant decrease of Na^+^ ions. These mechanisms are achieved by different classes of membrane proteins such as ion pumps, transporters, and channels [40]. Besides these genes, it was reported during the last decade that plants have protein homologs to the yeast plasma membrane protein 3 (PMP3) [41]. Numerous genes belonging to the PMP3 family have been isolated and characterized in several plants, including *Arabidopsis*, rice, wheat, barley, maize, sheep-grass, alkali-grass, and plantain [3,13,23,37,38,42]. These reports indicated that plant PMP3s might have a conserved function since the heterologous expression of some of them can functionally complement yeast pmp3 mutants by restoring the plasma membrane potential as well as the ion homeostasis [3,41]. We reported here the first isolation of a *TMP1* gene from the C4 halophyte grass *A. littoralis* (*AlTMP1*) and established its role in conferring abiotic stress tolerance. The predicted AlTMP1 protein belongs to the yeast plasma membrane proteins 3 (PMP3) and has two homologs sharing 87.7% and 86.4% amino acid identity with rice OsLTI6b and maize ZmPMP3.1, respectively. The isolated gene *AlTMP1* is induced by low temperature, salt stress, exogenous ABA, and osmotic stress. The rapid accumulation of *AlTMP1* transcripts suggests that this gene is involved in the early response mechanisms to abiotic stresses. These results are in the same line with reports in other plants showing that the expression of PMP3 homologs is also up-regulated by different abiotic stress stimuli [3,18,20]. In *A. littoralis*, *AlTMP1* was induced very early following cold, salt, ABA, and PEG treatments. A similar response was reported for *PutPMP3.1/2*, which are induced by different stresses, especially low temperature, salinity, and dehydration [23]. *ESI3* from wheatgrass is induced by NaCl, osmotic stress, and ABA treatments [37]. Similarly, it has been shown that the expression of *AcPMP3.1* and *AcPMP3.2* in sheep grass is induced in response to ABA, H_2_O_2_, SA, cold, salt and drought treatments [20]. Furthermore, it is known that plants can adjust abscisic acid (ABA) levels constantly in response to abiotic stresses, which in turn causes stomatal closure and induces expression of stress-related genes [43]. In this study, we showed that *AlTMP1* is regulated by ABA, suggesting that it may be involved in the ABA-dependent signaling pathway. The *AlTMP1* gene is induced by extracellular ABA treatment, whereas its nearest homolog gene *ZmPMP3.1* (86.4%) from maize is not [3]. Moreover, *AlTMP1* is highly induced by osmotic stress, while *ZmPMP3.1* is weakly induced by drought. In the same way for the second nearest homolog gene *OsLTI6b* (87.7%) it was shown that its transcripts accumulated very quickly in leaves after one hour of ABA or dehydration treatments then dropped to almost a basal level. These results indicate that the three genes have different expression patterns in response to ABA and drought treatments despite their high identity. This may be ascribed to differences in regulatory sequences and may reflect the fact that *A. littoralis* is a halophyte whereas maize and rice are glycophytes.

Similar to plant PMP3 homologs, *AlTMP1* protein has two putative hydrophobic transmembrane domains, localizing it to the plasma membrane and separated by a predicted short loop, which orientates both the N- and C-termini towards the apoplast. In addition, the subcellular localization assay of the GFP::AlTMP1 fusion confirmed a plasma membrane localization of AlTMP1. Similar results were also previously observed in wheat [17], maize [3], *Arabidopsis* [41], alkali grass *P. tenuiflora* [23] and *A. chinense* [20]. The association of PMP3 proteins with the plasma membrane corresponds to an expected role of PMP3s in regulating intracellular cation accumulation.

Accumulation of *AlTMP1* transcripts in transgenic tobacco conferred tolerance to NaCl, ionic, osmotic, cold and heat stresses under in vitro conditions. Furthermore, *AlTMP1* homozygous transgenic lines were able to grow until maturity stage, to set flowers and to produce seeds when grown in the greenhouse under continuous salt and drought stresses. Under similar growth conditions, NT plants exhibited a stunted phenotype. Both in vitro and greenhouse assays revealed that protection afforded by *AlTMP1* expression was more important for osmotic stress than for salt stress. Contrastingly, *OsLti6b* over-expressing rice plants exhibited no significant difference over non-transgenic controls under either salt or drought stress [27]. Accumulation of *ZmPMP3.1* in *Arabidopsis* conferred salt stress tolerance, but these transgenic plants were not evaluated for drought stress tolerance [3]. The enhanced tolerance observed in transgenic *AlTMP1* plants could be related to the low and high levels of accumulation in young tissues of Na^+^ and K^+^, respectively. The adverse effect of salinity on plant growth and yield is known to be alleviated when K^+^ and Ca^2+^ accumulate at a high level in plant tissues [44] thereby protecting young leaves from Na^+^ ion toxicity. This could explain the relative normal growth of *AlTMP1* plants and their ability to set and produce seeds under salt stress. In addition, the RWC of leaves was more important in *AlTMP1* plants than in NT under stress most probably due to an improvement in membrane stability. Indeed, the *AlTMP1* transgenic lines showed significantly higher MSI and considerably reduced electrolyte leakage under stress conditions compared to NT plants. These results were consolidated by the observation of a higher level of transcript accumulation of some stress-related genes (*NHX1*, *CAT1*, *APX1* and *DREB1A*) in the transgenic plants than in the NT. Taken together, these results can suggest that expression of *AlTMP1* can indirectly activate some stress-related genes leading to the observed tolerance to salt and drought stresses. Indeed, the up-regulation of *CAT1* and *APX1* genes in *AlTMP1* transgenic lines under both control and stress conditions could increase the capacity of the plant to alleviate the reactive oxygen species (ROS) produced by the plant facing salinity or drought. *HKT1* and *KT1* genes were down-regulated in transgenic plants. Since KT and HKT-type proteins are probable candidates promoting Na^+^ uptake into the root [45], their down-regulation could explain the lower Na^+^ concentration in transgenic plant tissues. The vacuolar Na^+^/H^+^ antiporter (*NHX1*) has long been proposed to play important roles in salt tolerance by the compartmentalization of Na^+^ into the vacuole [46]. This mechanism reduces the deleterious effects of Na^+^ excess in the cytosol and thus enhances water uptake and salt tolerance in plants by maintaining osmotic balance without spending much energy. DREB1A is reported to activate the transcription of more than 40 stress-inducible genes in *Arabidopsis* [47,48]. Based on these results, we propose that expression of *AlTMP1* may up-regulate *DREB1A* which in turn induce some of its target stress genes in transgenic tobacco and thus result in enhanced stress tolerance.

## 4. Materials and Methods

### 4.1. Plant Materials and AlTMP1 Gene Isolation

*A. littoralis* and *Nicotiana tabacum* var. *Xanthi* was used in this study. Seeds of an *A. littoralis* were collected from salt marshes near “Sfax” a coastal town in the middle of Tunisia.

A full-length cDNA corresponding to *AlTMP1* was isolated by screening an SSH cDNA library (cloned in pDNR-LIB vector, CLONTECH) previously prepared using RNA extracted from stressed *A. littoralis* roots during 15 days with 300 mM NaCl as described [31]. The differentially expressed full-length cDNA was sequenced by bidirectional sequencing with M13F and M13R primers (Table S1) and an ABI 3100 automatic DNA sequencer (Applied Biosystems, Foster City, CA, USA). The two sequenced fragments from 5′ and 3′, direction were assembled to generate the full-length cDNA “*AlTMP1*” sequence which was deposited in the Genbank with the accession number KY321744. The primers AlT1F5′ and AlT1R3′ (Table S1) were used to amplify the genomic clone of *AlTMP1* gene. The generated fragment of 623 bp was cloned into the pGEMT-easy vector (Promega, Madison, WI, USA), and three different positive clones were sequenced using SP6 and T7 universal primers.

The blastx and blastn were conducted by using the sequenced cDNA as a query to explore the homologs genes to *AlTMP1* in NCBI GenBank (http://www.ncbi.nlm.nih.gov). The orthologues genes were used to perform multiple sequence alignments, and the degree of amino acid sequence identity was determined by the sequence editor software BioEdit 7.2.5. To predict the phylogenetic relationships between *AlTMP1* and others PMP3 family proteins, we have used the MEGA6 software [49] to construct a phylogenetic tree. Various tools from Expasy (Expert Protein Analysis System, http://www.expasy.org/tools) were used to deduce the translated product and compute theoretical isoelectric point (pI) and molecular weight. The putative domains were identified by using the InterProscan search (http://www.ebi.ac.uk/interproscan). The prediction of the transmembrane helices of AlTMP1 putative protein was made via the THMM server v.2.0 (http://www.cbs.dtu.dk/services/TMHMM/).

### 4.2. Subcellular Localization of GFP::AlTMP1 Fusion Protein

The *AlTMP1* coding sequence was amplified by PCR using AlTMP1-B and AlTMP1-X primers containing *Bam*HI and *Xba*I restriction sites (Table S1). The amplified fragment was cloned into *Bam*HI/*Xba*I sites of the binary vector pCAMBIA2300-GFP to generate the fusion genes GFP::*AlTMP1* driven by the CaMV-35S promoter. Both constructs pCAMBIA2300-GFP::*AlTMP1* and pCAMBIA2300-GFP (as a control) were separately bombarded into a single layer of onion epidermal cells using the PDS-1000/He (Bio-Rad, Hercules, CA, USA) according to the manufacturer’s protocol [50]. After incubation on MS medium for 36 h at 28 °C in darkness, the GFP signal was monitored, and the microscope imaging was performed at Montpellier RIO Imaging Platform (http://www/mri/cnrs.fr) using a confocal microscope (LSM 510, Meta; Carl Zeiss MicroImaging, http://www.zeiss.de).

### 4.3. Q-PCR Analysis of AlTMP1 and Stress-Related Genes

Surface sterilized seeds of *A. littoralis* (1% sodium hypochlorite solution for 15 min, followed by washing six times with autoclaved distilled water, were germinated in Eppendorf tubes (Eppendorf, Hamburg, Germany) containing 500 µL half strength MS (Murashige and Skoog medium) [51] solid medium and grown as described by [31]. After two months of growth, plants were treated with different stress factors: high salinity (300 mM NaCl), high osmotic pressure (20% PEG 8000), low temperature (4 °C) and hormonal stress (100 µM abscisic acid (ABA)). Leaves from stressed plants were sampled at 0, 2, 4, 6, 24 and 48 h after each treatment and immediately frozen in liquid nitrogen and stored at −80 °C for RNA extraction and real-time quantitative RT-PCR (qRT-PCR) analysis of *AlTMP1* gene.

Total RNA was extracted from either *A. littoralis* or tobacco leaves using the RNAeasy Plant Mini kit (Qiagen, Hilden, Germany) according to the manufacturer’s protocol. The RNA was treated with DNaseI (RQ1 kit, Promega) at 37 °C for 15 min to remove the remaining genomic DNA. Synthesis of the first-strand cDNA was performed using 5 µg treated total RNA, SuperScript^TM^ III reverse transcriptase (Invitrogen, Carlsbad, CA, USA), oligo-(dT_18_) and random hexamer primers according to manufacturer’s instruction.

Quantitative real-time RT-PCR (qRT-PCR) was used for analyzing expression patterns of *AlTMP1* under various stress treatments in *A. littoralis* using qAlT1-F and qAlT1-R primers (Table S1). In addition, the transcript accumulation was monitored by real-time qPCR for eight stress-related genes (Table S1) in T2 homozygous transgenic lines and NT tobacco plants sampled under both control and stress conditions. The cDNAs prepared as described above from *A. littoralis* or transgenic tobacco lines were used in real-time qPCR reactions. The qPCR reactions were carried out using the LightCycler480 System and the LightCycler^®^ 480 SYBR Green I Master (Roche, Mannheim, Germany) according to the supplier’s manuals. The PCR cycling conditions were as follows: 95 °C for 3 min, followed by 40 cycles of 95 °C for 20 s and 60 °C for 30 s and 72 °C for 1 min. A melting curve was routinely performed after 40 cycles to verify primer specificity. Fragments of 18S rRNA gene (600 pb, 18SF and 18SR) and actin gene (380 bp, ACT-F and ACT-R) were used as an internal reference for *A. littoralis* and tobacco plants respectively (Table S1). The stress related genes monitored in transgenic tobacco encode Na^+^ transporter (HKT1, AB061311) [52], K^+^ transmembrane transporter (*KT1*, NM001336238.1) [53], Na^+^/H^+^ antiporter (*SOS1*, AY785147) [54], Na^+^/H^+^ exchanger 1 (*NHX1*, JX987081.1) [28], serine/threonine protein kinase (*SOS2*, NM_122932.5) [55], DRE-binding proteins (*DREB1A*, AF300970) [56], catalase1 (*CAT1*, U93244.1) [57] and ascorbate peroxidase (*APX1*, AK061841). All primers were designed by Primer3Plus (http://primer3plus.com/cgi-bin/dev/primer3plus.cgi) [58]. The quantitative analysis was performed using the ΔΔ*C*_T_ comparative method (2^−∆∆*C*t^ method, in which *C*_T_ indicates cycle threshold) [59]. Statistical analysis was performed on ΔΔ*C*_t_. For the analysis of *AlTMP1* expression under various treatments, ∆∆*C*_t_ = (*C*_t AlTMP1_ − *C*_t 18s_)_Time *x*_ − (*C*_t AlTMP1_ − *C*_t 18s_)_Time 0_. Time *x* is any treated time point (2, 4, 6, 24 and 48 h), and Time 0 represents the untreated time (0 h). For the analysis of stress-related genes expression in transgenic lines and NT, ∆∆*C*_t_ = (*C*_t target gene_ − *C*_t actin_)_transgenic line_ − (*C*_t target gene_ − *C*_t actin_)_NT_. Relative expression ratios are reported from three independent experiments (three biological repetitions).

For semi-quantitative RT-PCR Polymerase master mix (Kapa Biosystems, Woburn, MA, USA) was used. 10 µL of the PCR product was separated on a 1% agarose gel to visualize the intensity of gene amplification. As an internal control, the primers ACT-F and ACT-R (Table S1) were used to amplify a fragment of actin gene (380 bp) as a control for equal cDNA loading. The experiment was repeated three times to ensure reproducibility.

### 4.4. Generation of Transgenic Tobacco Expressing AlTMP1

The plasmid pDNR-LIB was digested with *Xba*I and *Sma*I to release the cDNA fragment of *AlTMP1* gene, which was purified from the gel and finally cloned in the binary vector pCAMBIA1390 (CAMBIA, Canberra, Australia) under the control of the CaMV35S promoter and Nos-polyA terminator. The resulting pCAMBIA1390-*AlTMP1* construct was then mobilized into *Agrobacterium tumefaciens* strain EHA105 [60] by electroporation method [61]. *Agrobacterium*-mediated leaf disc transformation of tobacco was carried out per standard protocol [62]. Transgenic T0 plants were grown to maturity after selection on MS agar medium containing 50 mg/L Hygromycin B (Sigma-Aldrich, St. Louis, MO, USA). T1 seeds were again germinated on MS agar medium with Hygromycin B, and the resistant transgenic lines were grown to obtain the T2 homozygous lines used for further analysis. The presence and expression of *AlTMP1* were ascertained by PCR and RT-PCR analysis, respectively.

### 4.5. Evaluation of Transgenic Tobacco Tolerance to Abiotic Stresses

The *AlTMP1* transgenic tobacco plants (L2 and L3) of homozygous T2 generation were used in the subsequent abiotic stress assays. Seeds of non-transgenic (NT) and transgenic plants were surface sterilized and plated on MS0, MS0m (MS0 with 350 mM mannitol) and MS0s (MS0 with 200 mM NaCl). The plates (three replicates of 100 seeds) were placed in a growth chamber under a 16 h light/8 h dark cycle at 25 °C. The number of germinated seeds was scored every two days for three weeks. Germination was defined as a clear emergence of the radicle through the seed coat. To survey the effects of salt and osmotic stresses on root elongation of NT and T2 (L2 and L3) transgenic *AlTMP1* lines. The seeds were germinated in MS medium supplemented with 0, 100, 150 and 200 mM NaCl or 200, 300 and 400 mM Mannitol and grown for one month. Root length, for both transgenic and NT seedlings, were determined after four weeks of growth.

To evaluate the effect of osmotic (400 mM Mannitol), salt (200 mM NaCl) and ionic (100 mM LiCl) stresses on shoot elongation, and RWC of leaves under in vitro culture conditions, transgenic and NT seedlings were transferred to stressing media after ten days of germination on MS0. The shoot length, number of leaves and dry weight was monitored following one month of growth. 

Chlorophyll content was estimated using 10 leaf discs from non-transgenic and transgenic plants (one cm leaf disc) per treatment. These discs were floated on MS liquid medium containing 0, 700 mM NaCl, 400 mM LiCl, 300 mM H_2_O_2_ or 20% PEG for 72 h. The total chlorophyll content in each sample was calculated after extraction in aqueous 80% acetone using the following formulae [63] which express [Chl a], [Chl b] and [Chls a + b] in µg·mL^−1^: [Chl a] = 12.70 × *A*_663_ − 2.69 × *A*_645_, [Chl b] = 22.90 × *A*_645_ − 4.68 × *A*_663_, [Chls a + b] = 20.21 × *A*_645_ + 8.02 × *A*_663_. The *A*_663_ and *A*_645_ represent absorbance values read at 663 nm and 645 nm wavelengths, respectively.

For freezing and heat stresses, one-month-old seedlings of transgenic and NT plants grown in peat under greenhouse conditions were incubated at −20 or 55 °C for two hours. Plant survival and dry weights were determined after five days recovery in the greenhouse. 

To monitor the drought and salt effects under greenhouse conditions, seeds of homozygous transgenic lines (L2 and L3) and NT plants were germinated and grown on MS medium supplemented with 50 mg/L hygromycin for one month. The seedlings were then transferred to pots filled with a 3:1 mixture of soil and peat and grown in a greenhouse for more two weeks before being exposed to stress treatments. For normal growth conditions transgenic and NT plants were irrigated with water to maintain the RWC of soil at 75%. For salt stress treatment, the same irrigation program was used with the addition of 250 mM NaCl to water until the end of plant cycle. This NaCl concentration was increased gradually from 150 to 250 mM within the first 15 days of stress treatment. For drought stress, the RWC of the soil was maintained at 25%. For all treatments, leaf RWC, plant height, dry weight of roots and shoots, and seed yield were determined.

### 4.6. Ion Content Determination

The accumulation of Na^+^, K^+^ and Ca^2+^ concentrations in young and old leaves was determined for plants grown at 250 mM NaCl. Leaves were collected from salt-stressed treated plants and then washed thoroughly with distilled water, then dried at 80 °C until constant weight was reached. The dried leaves were incubated for one week with 0.5% HNO_3_. The filtrate from each sample was analyzed by atomic absorption spectrometer (Thermo Scientific™, Loughborough, UK). Concentrations were calculated as mg·g^−1^ dry weight. The K^+^/Na^+^ ratio was also determined.

### 4.7. Membrane Stability Index and Electrolyte Leakage Assay

Membrane stability index (MSI) was measured as described by [64]. Two sets of leaf samples were taken in 10 mL of double-distilled water. One set was heated at 40 °C for 30 min in a water bath, and its conductivity (*C*1) was recorded using Oakton PC2700 conductivity meter (Oakton Instruments, USA). The second set was boiled at 100 °C for 10 min before having its conductivity (*C*2) measured. MSI was calculated using the following formula: MSI = [1 − (*C*1/*C*2)] × 100.

Electrolyte leakage was measured as described by [65]. The third leaf from the top, with similar size, was collected from three plants for each line and each treatment and washed thoroughly with deionized water to remove electrolytes adhered on the surface. The samples were placed in closed vials containing 10 mL of deionized water, incubated at 25 °C on a rotary shaker for 24 h, and the electrical conductivity of the solution (*EC*1) was determined using an Oakton PC2700 conductivity meter (Oakton Instruments, Vernon Hills, IL, USA). The samples were then autoclaved at 120 °C for 20 min, and the final electrical conductivity (*EC*2) was obtained after cooling at 25 °C. The electrolyte leakage was defined as follows: Electrolyte leakage (%) = (*EC*1/*EC*2) × 100.

### 4.8. Statistical Analyses

The data for all experiments have been calculated from three replications, and different values have been presented as mean ± s.e. (standard error). Statistical analyses were carried out using xlSTAT, and one-way ANOVA [66] followed by Bonferroni’s *post hoc* test was used to assess if the variations between treatments are statistically significant. Mean values that were significantly different at *p* < 0.05 from each other are marked with different lowercase letters inside the figures.

## 5. Conclusions

In the present study, an *AlTMP1* gene was isolated from the halophyte C4 grass *A. littoralis*. This gene encodes a transmembrane polypeptide of 57 amino acids belonging to the family of plasma membrane protein 3 (PMP3) and has two nearest homologs in rice (*OsLTI6b*) and maize (*ZmPMP3.1*). This study is the first demonstration that a member of the PMP3 family confers stress tolerance and enhances seed yield in transgenic *AlTMP1* lines subjected to continuous drought and salinity treatments in the greenhouse. Noteworthy, this gene confers higher tolerance to drought than to salt stress. Finally, the expression of *AlTMP1* resulted in the deregulation of some stress related tobacco genes. Further study using RNAseq analysis of transgenic plants expressing *AlTMP1* will contribute to our understanding of the mechanisms behind the stress-tolerance observed in these plants.

## Supplementary Materials

Supplementary materials can be found at www.mdpi.com/1422-0067/18/4/692/s1.
