# Supplementary material for: Ectopic Expression of Aeluropus littoralis Plasma Membrane Protein Gene AlTMP1 Confers Abiotic Stress Tolerance in Transgenic Tobacco by Improving Water Status and Cation Homeostasis
Source: Int J Mol Sci. 2017 Mar 24;18(4):692. doi: 10.3390/ijms18040692 (PMC5412278; doi:10.3390/ijms18040692)
Supplement: Supplementary file 1 [file ijms-18-00692-s001.pdf]

Supplementary Materials: Ectopic Expression of *Aeluropus littoralis* Plasma Membrane Protein Gene *AlTMP1* Confers Abiotic Stress Tolerance in Transgenic Tobacco by Improving Water Status and Cation Homeostasis

Walid Ben Romdhane, Rania Ben-Saad, Donaldo Meynard, Jean-Luc Verdeil, Jalel Azaza, Nabil Zouari, Lotfi Fki, Emmanuel Guiderdoni, Abdullah Al-Doss [1] and Afif Hassairi

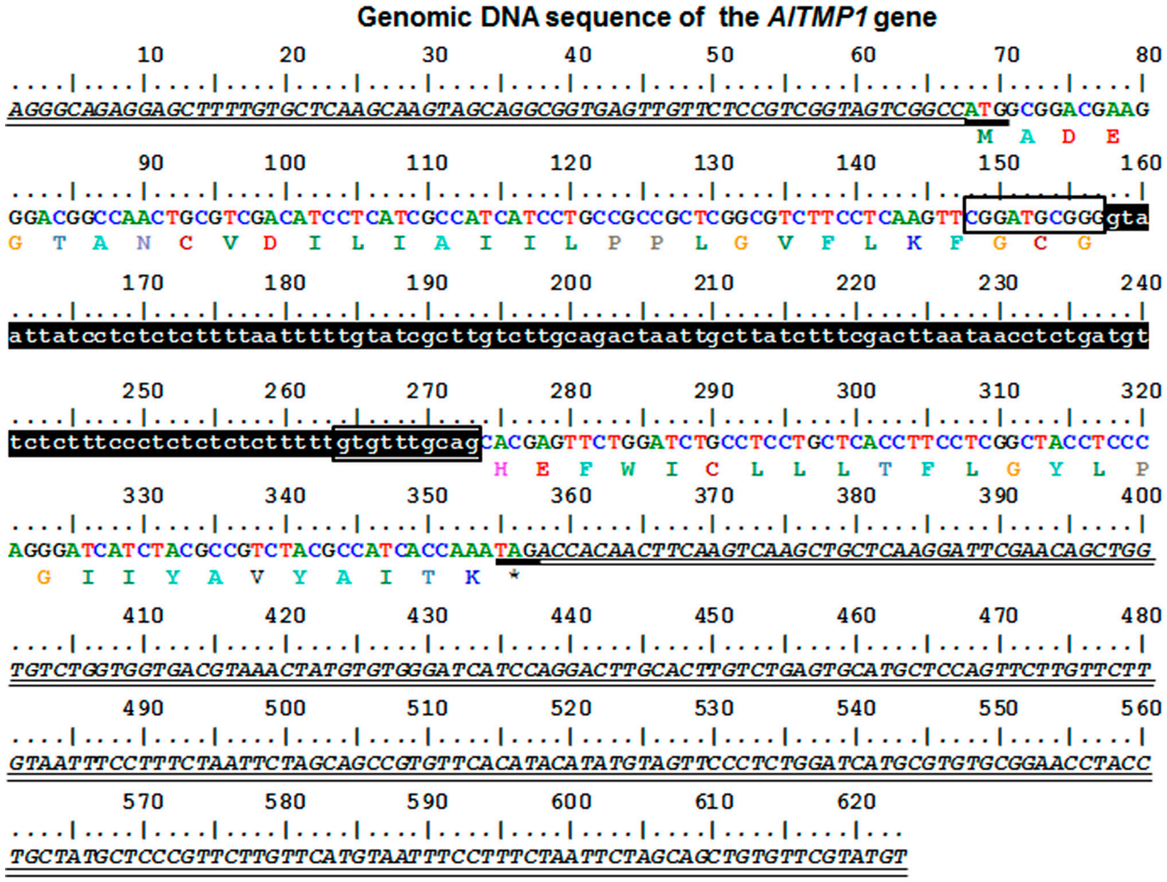

Figure S1. Genomic DNA sequence of *AlTMP1*. Translational start codon (ATG) and stop codon (TAG) are underlined. The 5′ and 3′ non-coding sequences are double underlined. Exon sequences are in uppercase letters, whereas intron sequences are in lowercase letters and shaded in black. The donor splice sites are boxed. The sequence was analyzed with NetGene2 Server.

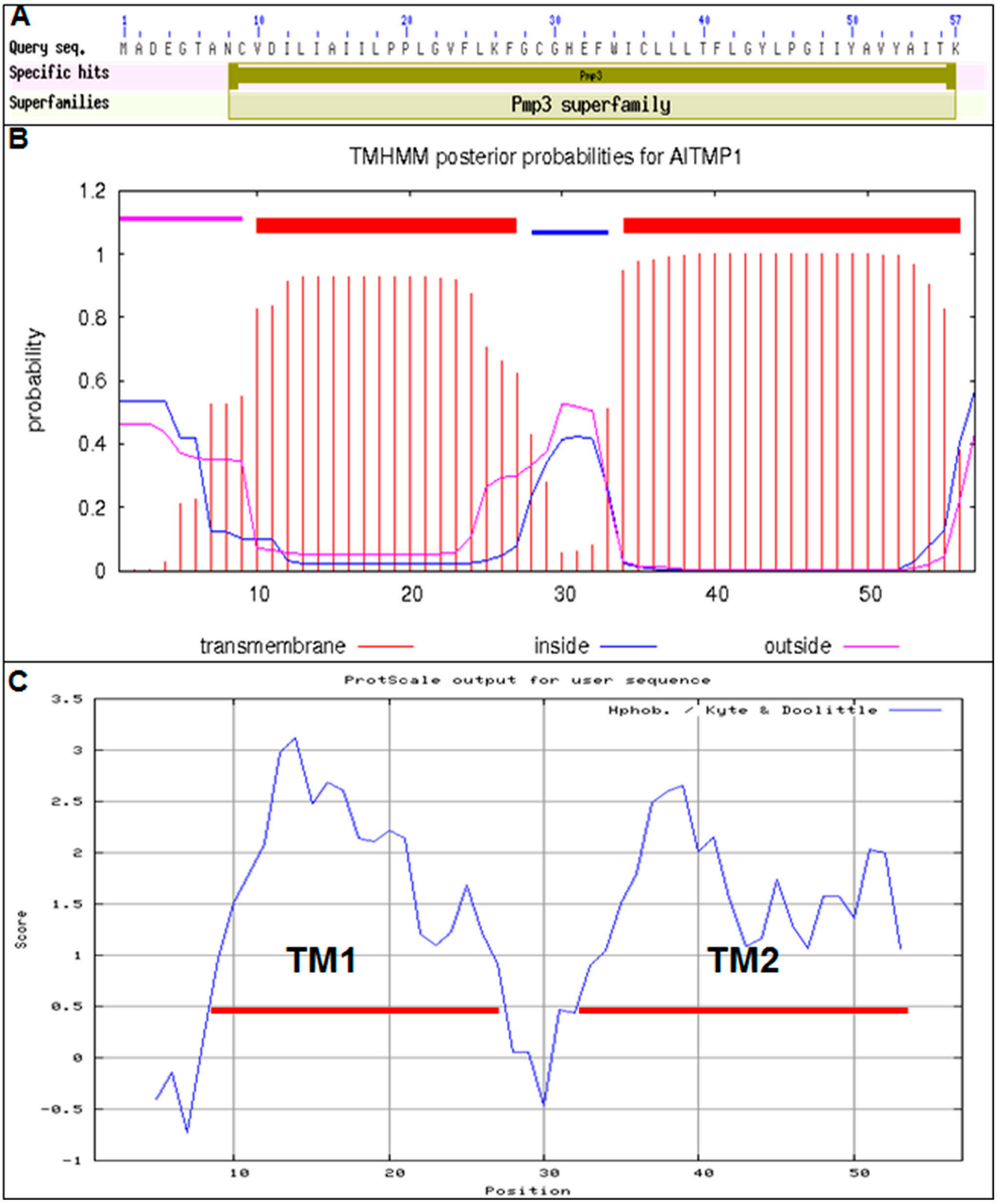

Figure S2. Prediction of conserved transmembrane domains and helices in AlTMP1. (**A**) The putative conserved domain of the AlTMP1 protein, Images were generated and taken from the NCBI BLASTp. (**B**) The transmembrane domains were predicted using the TMHMM Server v. 2.0. (**C**) Hydropathy Kyte and Doolittle profile for AlTMP1 protein.

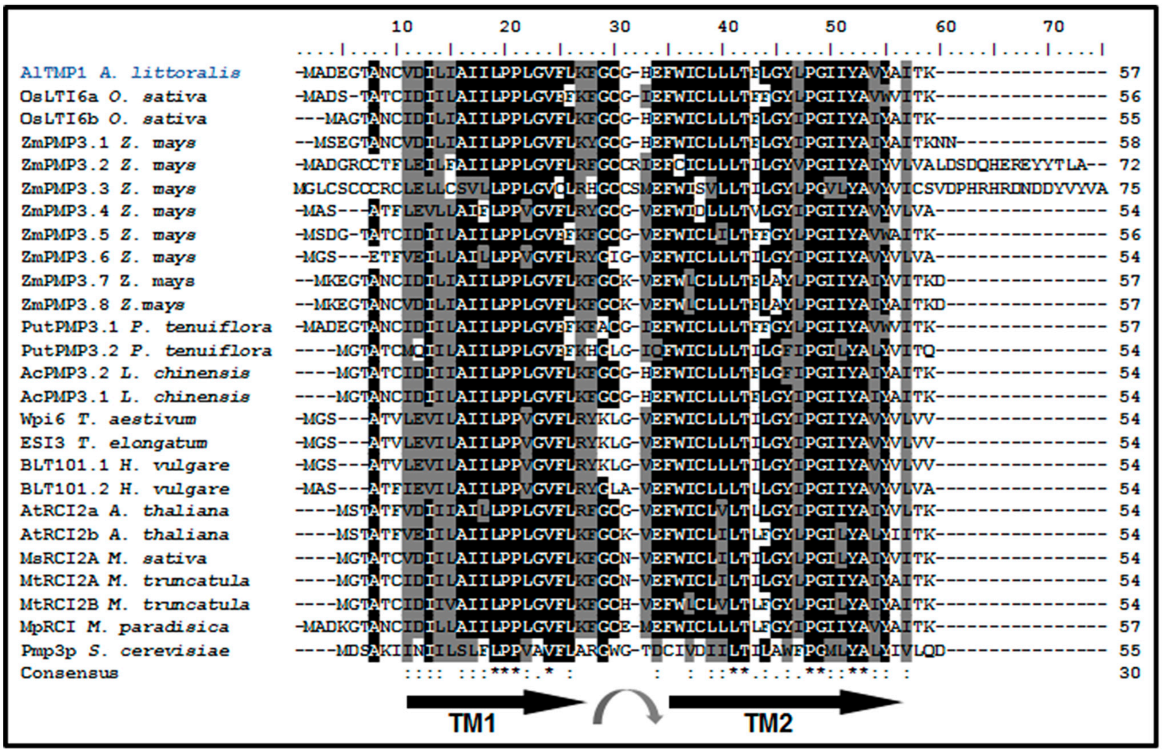

Figure S3. Amino acid sequence alignment analysis of AlTMP1 and other PMP3 proteins. The GenBank accession numbers of different PMP3 proteins used in generating the alignment are as follows: ZmPMP3.1 (NP_001107634), ZmPmP3.2 (ACG31120), ZmPmP3.3 (ACG34525), ZmPmP3.4 (ACG26760), ZmPmP3.5 (ACG47392), ZmPmP3.6 (ACG48459), ZmPmP3.7 (ACG27760), ZmPmP3.8 (NP-001151840); OsLTi6a (AAT37941), OsLTi6b (AAT37942); PutPMP3.1 (BAG54793), PutPMP3.2 (BAG54794); AtRCI2a (NP-187239), AtRCI2b (NP-187240); BLT101.1 (CAA80984.1), BLT101.2 (CAC37082); WPI6 (BAE07207); MpRCI (ACA66247); Pmp3p (EEU06338); ESI3 (P68178); MtRCI2A (AES82350), MtRCI2B (AES82353); MsRCI2A (AFI47457); AcPMP3.1 (BAD34658), AcPMP3.2 (BAD34659). The identical amino acids are shaded in black, while the amino acids with similarity larger than 75% are shaded in dark gray. Black and gray arrows represent the predicted transmembrane domains and the putative loop structure, respectively. Table S1. Sequences of primers used in PCR and qRT-PCR analysis.

Table S1. Sequences of primers used in PCR and qRT-PCR analysis.

Primer Name	Sequence 5′–3′
AlT1-F5′	AGGGCAGAGGAGCTTTTGTG
AlT1-R3′	AGCAGGTAGGTTCCGCACACG
qAlT1-F	AGTAAAGCTGCTCAAGGATTCG
qAlT1-R	ATGTATGTGAACACGGCTGCTA
AlTMP1-B	AGTCCTGGATCCAGGGCAGAGGAGCTTTTGTG
AlTMP1-X	AGTCCTTCTAGACAGAGGGAACTACATATGTAT
18S-F	GCAAGTCTGGTGCCAGCAGCC
18S-R	CTTCCGTCAATTCTTTTAAG
ACTF	GTGCCCATTTACGAACGATA
ACTR	GAAGACTCCATGCCGATCAT
M13F	GTAAAACGACGGCCAGT
M13R	AAACAGCTATGACCATGTTCA
qHKT1-F	CATTGTTCGGTGCTGTCGAC
qHKT1-R	AATGCCAAGTTCTGCACCAC
qKT1-F	G ACGGGAGTTCCAGCTGTTT
qKT1-R	TCCCGGTTCGGTTTCAACG
qSOS1-F	TATCAGGTGGAGGCTAGAGC
qSOS1-R	TCATGCTCCCGTACATGCTC
qNHX1-F	TTGATGAGAGGCGCAGTGTC
qNHX1-R	TTGACTGGCTAGAAGTGGCG
qSOS2-F	GAGGGCAACAGAAGGAATAC
qSOS2-R	GAACAGAGGGATCCAAACCA
qDREB1A-F	ACTGGACGTCCTGAGTGACA
qDREB1A-R	GGCATCGGAAGCCAGAAAAG
qCAT1-F	TGCTCCAAAGTGTGCTCATC
qCAT1-R	GAAGCAAGCTTTTGACCCAG
qAPX1-F	AACGTTTGGGCTTTTCTCCT
qAPX1-R	TCAACAGCAACAACTCCAGC
